# The identity of clinical associates in psychology: a cross sectional, national survey

**DOI:** 10.1186/s12909-024-05802-7

**Published:** 2024-07-31

**Authors:** Ciarán O’Driscoll, Kiana Azmoodeh, Ravinder Rana, Gillian Hardy

**Affiliations:** 1 CORE Data Lab, Centre for Outcomes Research and Effectiveness (CORE), Research Department of Clinical, Educational, and Health Psychology, UCL, London, UK; 2 East London NHS Foundation Trust, London, UK; 3 Centre for Psychological Services Research, Department of Psychology, University of Sheffield, Sheffield, UK

**Keywords:** Clinical associate in psychology, Psychological training, Qualitative survey, Psychologists

## Abstract

**Background:**

The Clinical Associate in Psychology (CAP) is a new psychological profession within the National Health Service (NHS) in the United Kingdom. This paper considers the processes developing the CAPs’ professional identity, specifically how their roles are embedded within services.

**Methods:**

This study utilised an online survey of CAPs and all academic, clinical and managerial staff involved with CAPs. An inductive thematic analysis was undertaken.

**Results:**

A total of 164 participants responded to the survey. Five themes were identified: Widening Access to Psychology, Workforce Development, Navigating the Unfamiliar, Trained [Master’s level] Professionals and An Emerging Ethos. In addition, key skills and unique contributions from CAPs were identified.

**Conclusions:**

A clear professional identity is emerging, with CAPs depicted as offering versatile interventions in diverse health care settings, fostering a positive and encouraging integration of psychological expertise into the healthcare service. The study highlights areas for development to facilitate the growth and advancement of the role within the psychological workforce.

**Supplementary Information:**

The online version contains supplementary material available at 10.1186/s12909-024-05802-7.

## Introduction

The Clinical Associate in Psychology (CAP) is a new psychological profession within the National Health Service (NHS) in the United Kingdom. The Clinical Associate in Psychology apprenticeship model started in 2020. During this period, the Community Mental Health Transformation Programme was being implemented as part of the NHS Long Term plan [1], placing a strong emphasis on patient-centric care, integration, and population health perspectives. With increasing demand and a limited supply of applied psychologists nationwide, this initiative sought to expand the highly skilled psychological workforce across 12 pilot NHS sites, leading a ‘transformation’ in mental healthcare^1^.

The Clinical Associate in Psychology (CAP) role sits within the family of psychological professions, a relatively new grouping of different professions, which means that alongside CAPs developing their own professional identity, the role itself is in the process of being defined. The process through which a job takes on a professional identity includes the development of (1) a code of practice, (2) recognised educational training, (3) a professional body, (4) self-regulation, (5) a defined set of specialised skills and (6) recognition by others [3].

Professional identity is seen as core to the development of job satisfaction, staff retention, multidisciplinary work, and the quality of patient care [4, 5]. They defined professional identity as a person’s self-perception based on their values, motives, experiences, attributes, and beliefs related to their profession. Cornett, Palermo, and Ash [6] identified five major themes important to professional identify from the health professions literature: ‘The lived experience of professional identity’ (the practice and consolidation of skills), ‘The world around me’ (workplace and organisation influences), ‘Belonging’ (the profession in relation to other professions), ‘Me’ (personal identity and self in relation to others), and ‘Learning and Qualifications’ (qualifications and learning opportunities). These themes were developed primarily from nursing and medical studies, as there is sparse literature investigating other professions. It is not known, therefore, whether similar themes would be generated from the psychological professions and from new professions.

In England, CAPs undergo 18 month, postgraduate level training (MSc) and receive professional recognition through the British Psychological Society (BPS). The BPS also provides CAPs with a code of practice and standards for training [7]. This means that the first four requirements of a job moving to taking on a professional identity are met. Even when these institutional markers of a profession are in place, there remains the necessary task of public recognition before the profession is fully established.

This paper considers how CAPs experience developing their own professional identity and the processes of professionalism, specifically how CAP roles are embedded within services. As a new and emerging occupation, it is important to establish the standards and expectations for the role. The aim of this study was therefore to gather feedback about the current implementation of the role and identify areas for improvement by surveying all individuals involved with CAPs. This can inform our efforts to innovate and enhance the role of CAPs, potentially leading to improved professional identity and contribution to patient care.

## Method

### Design

This study utilised an online survey of CAPs and all academic, clinical and managerial staff involved with CAPs. Ethical approved was obtained from the University ethics committee (reference number: 21883.003 Qualtrics was utilized for collecting survey data, while NVivo was utilized as a coding software for qualitative data analysis.

### Participants

The participants in this study were representatives from educational providers (lecturers and programme directors), heads of psychological services within NHS Trusts, practitioner psychologists supervising CAPs, and qualified and apprentice CAPs. All participants were from England and were recruited through university providers. To ensure adequate coverage, educational providers sent out an email inviting them to participate in the study to their staff, apprentices, heads of services and clinical supervisors, who in turn were asked to disseminate within their networks. The email included a link to the online survey. Informed consent was obtained from all participants prior to commencing the survey. Participants from Scotland and Wales were not included as their models of training are different.

Participants were recruited from 11 of the 12 affiliated universities (*N* = 164). The job roles and CAP work setting of the participants are provided in Table 1.

**Table 1:** Demographics of respondents

Affiliated University	*N* = 164	
University of Exeter	31	19%
Keele University	2	1%
Nottingham Trent University	8	5%
University of Plymouth	26	16%
Royal Holloway University London	1	1%
University College London	49	30%
University of East Anglia	25	15%
University of Essex	8	5%
University of Kent	7	4%
University of Sheffield	5	3%
**Job Role**		
CAP apprentice	79	49%
Clinical Supervisor	22	14%
Lecturer	12	7%
Programme Director	5	3%
Psychology Lead	16	10%
Qualified CAP	28	17%
**CAP work setting**		
Acute Inpatient	8	8%
Adult CMHT	59	57%
CAMHS	12	12%
Crisis Team/HTT	6	6%
Forensic Services - Low Secure	1	1%
Health	2	2%
Older Adult Community	10	10%
Primary Care	6	6%

CMHT = community mental health team; CAMHS = child and adolescent mental health service; HTT = home treatment team

### Questionnaire

A questionnaire (see Supplementary Material) was used to gain information from a large number of respondents and enabled the collection and analysis of qualitative data. The survey consisted of a series of questions about the participants’ experiences of working with or as CAPs. While we aimed to collect a ‘wide-angle lens’ on the topic [8], qualitative survey data can sometimes be slightly ‘thinner’ than methods such as interviews. We attempted to alleviate this through use of the grid elaboration method [9]. The grid elaboration method starts with each participant’s unique associations to the question. To elicit participants’ initial associations, they were asked to provide a word or phrase they associate with the CAPs role in four separate boxes. Participants were then asked to elaborate in as much detail as possible on these associations. Following these questions, specific questions relating to the CAP role were asked to ensure coverage of the features of the role.

### Data analysis

Codebook Thematic Analysis [8] was used, following steps of Template Analysis (Brooks et al., 2015). A codebook was developed and two raters were designated to guide the analysis, balancing reflexive aspects with positivist practices in qualitative research to address researcher bias. This method is neither entirely positivist nor reflexive, but can be used flexibly with a “subtle realist” approach to address specific research questions. Context and perspective are acknowledged as influencing factors while seeking to access underlying phenomena [10]. We used an inductive approach to develop codes and themes from the data content, which we revised and clustered into themes to best characterise the data. We used data extracts to exemplify themes and discuss salient features in more detail.

Reliability and validity was established through the credibility, transferability, dependability, and conformability [11]. Credibility: members of our research team have extensive experience in delivering clinical training, practicing as clinicians, and organisational management. Transferability: individuals were purposively selected for the study to represent the range of professionals with insight into the CAP role Dependability: guidelines and procedures were put in place and followed. Both the first and second authors familiarised themselves with the dataset by reading each participant’s responses. The initial codebook was then generated by the first author and applied to the data. The second author assessed 25% of the data units to examine the codes generated, and the two authors refined and agreed on the final codebook and its application to the dataset. Both coders developed the thematic structure together through mutual agreement, discussing and refining themes based on the data and the coding. Conformability: Both coders work on a CAP programme delivering training and are clinical psychologists; therefore, our own biases and assumptions may have influenced the research process. We tried to mitigate these biases and assumptions by being as open-minded as possible during the coding and being blind to the details about the participants (their role, setting, associated university and Trust) during the coding and theme development.

## Results

### Participants

One hundred and sixty-four people completed the qualitative survey. The demographics of the participants are presented in Table 1.

### Thematic analysis

A thematic structure was devised, drawing together the salient themes and subthemes identified among the data (see Table 2). The five superordinate themes are discussed below with a narrative summary of each, including a discussion of subthemes and links between themes alongside illustrative quotes.

**Table 2:** Themes

**1. Widening access to psychology**
1.1 For Staff - A Presence of Psychology in Teams
1.2 For Clients - By Bridging the Gap
1.3 For CAPs - Providing a Route into the Profession
**2. Workforce Development**
2.1 Diversity
2.2 Creative use of resources
2.3 Flexible and Versatile
2.4 Supervision
**3. Navigating the Unfamiliar**
3.1 Misunderstood
3.2 The New and Uncertain
3.3. Career Progression
3.4 A Compass for the Future
**4. Trained [Masters-Level] Professionals**
4.1 Core Psychological Skills
4.2 A Broad, Yet Specialist, Provision
4.3 Responsibility
4.4 The Demands of Apprenticeship Training
**5. An Emerging Ethos**
5.1 Being with Clients
5.2 Commitment to Development
5.3 Professionalism
5.4 Considering the System

#### Widening Access to psychology

This theme broadly captures three distinct subthemes, encompassing the widening of access to psychology for: Staff – A Presence of Psychology in Teams (subtheme 1.1), for Clients – By Bridging the Gap (subtheme 1.2), and for CAPs - Providing a Route into the Profession (subtheme 1.3). For staff, the CAP apprentices were described as providing a presence of psychology in teams, offering access to psychologically informed thinking, formulation and reflective spaces. The ‘front-line’ nature of the role was highlighted, with CAPs integrated within teams providing a psychological perspective and support to staff. The CAPs were also described as ‘sharing the load’ with practitioner psychologists.

*“Service leads and supervisors who I have discussed CAPs report that having a CAP in their service has directly increased the psychological thinking skills of the wider team*,* from support workers to nurses and OTs [occupational therapists]*, etc.,* through offering formal consultations and supervision support but also through corridor conversations and building strong relationships with the teams.” (161 - Lecturer)*.

*“We have been able to create the role to fit the need in services. We are able to fill gaps in service provision by offering talking therapies at the “front door” (on referral) and by creating new groups and programmes” (78 - CAP).*

*“allows for more patients to be seen (as those that can be helped by a CAP are*,* and those who need a CP thus wait for less time)” (158 – Psychology Lead)*.

For clients, CAPs were reported to bridge a number of ‘gaps’ within service provision. For some, this was characterised by CAPs being positioned and available to offer timely psychological support and, in some cases, offering interventions that may not have been available before. CAPs were described as providing support for those who would otherwise not meet the threshold criteria for secondary care or specialist therapy services (while being unable to benefit from primary care services).

*“Way of operating as a service and that CAPs offers an applied*,* formulation driven development opportunity that is not model specific for people who might have been lost otherwise.” (11 – Programme Director)*.

*“CAPs are the first people to provide short term intervention in our service and also run groups” (82 - CAP)*.

Finally, the role was considered to improve access to a career in psychology for CAPs, an otherwise challenging and restricted career path. The role was said to sit between a nonqualified Assistant Psychologist and a registered Practitioner Psychologist. This theme links closely with Theme 4, which positions CAPs as Trained [Masters-Level] Professionals. Career access was broadly considered through two routes. For some, the CAP role was pursued or experienced as a ‘stepping stone’ towards doctoral training, offering movement towards further qualification and professional aspirations. Another perspective was that the role offered an alternative route into the wider applied psychology and mental health profession, allowing individuals who may not want to, or may have struggled to, pursue doctoral level training and join the workforce.

*“Some are also now moving into management positions*,* which have also been hard to fill but they are doing well in.” (12 – Psychology Lead)*.

#### Workforce development

This theme broadly spoke of the CAP role bringing about a shift within the psychology profession, encompassing strategic benefits as well as innovative transformation. Diversity was identified as a subtheme (2.1), with extracts highlighting hopes and a view that CAPs bring a wider breadth of experiences based on aspects of their own identities, strengthening service provision through their offer of new and important alternative perspectives while improving access for clients whose engagement may be affected through not otherwise seeing themselves represented in the workforce. This links closely with Theme 1, with diversity discussed as both widening access for clients (subtheme 1.2) and enabling recruitment of individuals historically lost through barriers in the system, thus widening access for CAPs (subtheme 1.3).

*“They bring difference in lots of different ways in recruiting people that would be lost to health services more generally. They also bring a different way of operating*,* allowing services to be more psychologically informed in their practice.” (10 – Programme Director)*.

Respondents highlighted that the CAPs role represented a Creative Use of Resources (subtheme 2.2), for instance, through offering cost-savings for underfunded services and through offering services the chance to provide bespoke on-the-job training that is driven by and tailored to local needs.

*“The apprenticeship route allows providers to use their apprenticeship levy for training*,* which is highly cost efficient. Apprentices provide a consistent ongoing service while training which services can rely on. Apprentices are more likely to stay in service post qualification*,* which makes the investment in their training even better value.” (154 – Psychology Lead)*.

The role was thought to be Flexible and Versatile (subtheme 2.3), with specific references made to the CAPs being able to work with varying complexities in client presentation and need, alongside systemic challenges, particularly the transformation of services developing new models of care. The flexibility of the role was seen as fostering innovation and the ability to do things differently, for instance, community psychology approaches. Both the remit of the role and the ability to work through formulation were considered important features supporting the ability to be flexible and versatile.

*“Having worked as an assistant psychologist and PWP there were very defined parameters around my role and the type of work I would assess*,* formulation and offer interventions. As a CAP I feel there is greater flexibility in the role to work creatively ensuring psychological approaches are bespoke to the client.” (71 - CAP)*.

Finally, Supervision (subtheme 2.4) was identified as a subtheme that captures some of the challenges faced by services, supervisors and CAPs alike when considering CAP apprentices as part of the workforce. Some respondents expressed concerns regarding the time required by supervisors to support apprenticeship learning. Conversely, in the qualified CAP role, some said that supervisor availability was limited, voicing a need for support and containment, with risk of isolation and unreasonable expectations being placed on CAPs (related to subtheme 3.2)

*“Not enough qualified staff in some services leading to more autonomy rather than supervised practice” (103 - Lecturer)*.

#### Navigating the unfamiliar

Consistently, respondents referred to the emerging nature of the CAP role and thus an inherent unfamiliarity. One manifestation of this was that the CAP role was described to be at times Misunderstood (subtheme 3.1), with staff teams struggling to distinguish between CAPs and other members of the psychology workforce. At times, CAPs themselves could not see a distinction between their own role and that of a practitioner psychologist. It was feared that expectations were being placed on CAPS that were outside of (i.e., both above and below) the remit of their role and responsibilities.

*“There is a real lack of understanding around our role*,* despite lots of work to try and integrate ourselves - perhaps because there has been confusion amongst ourselves about our exact remit” (78 - CAP)*.

*“The common risk will be a professional dissatisfaction and/or burnout resulting from working with a role that is as yet poorly understood in the healthcare economy*,* and may place inappropriate expectations upon them.” (151 – Programme Director)*.

The novelty of the role was highlighted, with it characterised as New and Uncertain (subtheme 3.2) across a number of domains. First, in terms of what staff teams might expect CAPs to bring to services, second, in terms of what CAPs could expect by way of job plans and support in the post, and third, with regard to future prospects and professional identity. That said, this ‘newness’ was also praised for its corresponding opportunities, with an enthusiasm brought by CAPs highlighted as a welcome and necessary “energy” within services.

*“The CAP role can look extremely different depending not only on the service or Trust*,* but between the same services within a Trust” (78 – Psychology Lead)*.

A key component of the reported unfamiliarity was Career Progression (3.3), with the role perceived to have an undefined developmental trajectory. Concern was raised regarding a lack of (current) professional registration, which could be experienced as invalidating the position of CAPs among the wider profession. Development opportunities were reported to be unknown, and progression unclear. This narrative was contrasted against examples of development on a case-by-case basis, including individuals working towards leadership roles, offering possibilities and hope.

*“… for other professions it is much easier to develop than it is for CAPs at the moment - something that comes down to not having a registration body and not being recognised as a core profession.” (16 – CAP apprentice)*.

With challenges and concerns noted, respondents offered ideas and direction for the CAP role, providing a Compass for the Future (subtheme 3.4). Establishing a professional identity with clear prospects was consistently suggested, with extracts speaking to the provision of a clear career framework and national strategy, progression within Trusts for qualified CAPs, posts built into staff teams, and stories of CAP roles post qualification to characterise a growing collective voice. In response to uncertainty regarding the CAP role, there were suggestions for greater clarification of CAP responsibilities and greater awareness and training among supervisors and teams.

*“Lack of a clear career pathway meaning to progress CAPs either have to go into Clinpsyd {doctoral level training] or have to become general managers within health care.” (4 – Psychology Lead)*.

#### Trained [Masters-Level] professionals

There was an emphasis on CAPs as Trained Professionals, with an MSc level professional qualification. This theme highlighted their Core Psychological Skills (subtheme 4.1), which span a breadth of areas of psychological practice. Given the brevity and specificity of the responses related to core skills, these themes have been formatted into a list. The core skills include assessment; psychological formulation; intervention (emphasis on practical intervention); drawing on knowledge of psychological theories and models; employing empathy and compassion; research and service evaluation skills; working with autonomy and decision-making; communication: interpersonal and listening skills; ability to work with teams and provide supervision/training; understanding of mental health difficulties and risk management; being flexible and adaptable; working with professionalism and ethical practice; working with communities; organisational and time management skills; and critical thinking and problem-solving abilities.

In contrast to other psychological professions, CAPs were reported to offer A Broad, Yet Specialist, Provision (subtheme 4.2), which allowed for training that offered a breadth of knowledge while developing specialist skills and tailored expertise according to the service area and local needs. However, for some, it was not broad enough, and for others, it was not specialised enough (e.g., more modality-specific training).

*“I think the CAP role offers depth of practise through the application of psychological thinking to a particular area or group. It moves away from the assumption that as a Clinical psychologist*,* Occupational therapist*,* nurse or social worker that you can work with any group/area following limited placement experience during your training.” (72 - CAP)*.

Examples were provided of the numerous ways in which CAPs have been holding Responsibility (subtheme 4.3) as trained professionals, with descriptions of CAPs as valued and competent members of teams. Their growing autonomy was highlighted as independent practitioners working effectively under supervision. There were concerns about the need for safeguards around responsibility due to possible misunderstandings about the role of the CAP and overzealous expectations, in line with subtheme 3.1 (Misunderstood).

*“They are the one’s running the main interventions the service provides” (49 CAP apprentice)*.

*“There is also a risk of them being pressured to take on more therapy-esque work than they are trained to*,* to take on parts of a CP’s role that they are not equipped to or under supervised or supported” (45 – Lecturer)*.

Whilst the training component of the CAP apprenticeship and role was largely welcomed for its ability to meet CAP, client and service needs, the nature of said training was discussed and The Demands of Apprenticeship Training (subtheme 4.4) noted. In particular, responses highlighted the challenge of balancing multiple demands, i.e., academic learning, assignments, and the development of clinical competencies, against a backdrop of personal circumstances and efforts to maintain wellbeing. There was a call for examination of the training experience, encompassing national consistency amongst training programmes, clear guidance and joint communication between employers and training providers, and a review of teaching content and assignments to best reflect learning that supports the delivery of the role. For instance, ensuring matching between the curriculum and employer needs, in some cases, supervisors consider themselves to be delivering a lot of teaching, requests for face-to-face teaching (where online is provided) and refining of the knowledge, skills and behaviours (KSBs) associated with the apprenticeship.

*“From a trainee perspective*,* there is an awful lot to learn and limited support. Learning on the job with real clients from the beginning makes you acutely aware of the need to get it right*,* and this brings with it some intense pressure.” (13 – CAP)*.

#### An emerging ethos

A final theme was identified relating to An Emerging Ethos (theme 5), capturing the perceived values, approach and core ideals of the CAP role. Where respondents noted the need for a professional identity, the foundations of this need were proposed. Four subordinate subthemes were generated, each outlined in turn.

Respondents spoke of an ethos of Being with Clients (subtheme 5.1). This captured a range of therapeutic process skills, including compassion, empathy and person-centred care. In keeping with subtheme 2.1 (Diversity), respondents highlighted an approach of respect, inclusivity and a championing of diversity through an emphasis on working as culturally competent practitioners and a valuing of lived experience.

*“a passion for developing relationships with clients*,* working alongside them rather than just for them*,* collaborating with clinical teams*,* and respecting clients.” (15 – CAP apprentice)*.

A clear Commitment to Development (subtheme 5.2) was outlined, emphasising an underlying curiosity and interest among CAPs. Through engaging with self-reflection and reflective practices, the CAPs’ willingness to learn and develop was highlighted. This stance and an application of hard work and dedication was commended, particularly within the context of challenging service contexts, with CAPs demonstrating both resilience and adaptability.

*“pioneers - being a new profession means we’re working in a way less established and I think takes a certain level of confidence / willingness that is needed” (110 – CAP).*

The role was characterised as being underpinned by Professionalism (subtheme 5.3), with knowledge and implementation of ethical principles readily put into practice and CAPs working with both responsibility and accountability. Related to theme 4, whereby CAPs were noted as Trained Professionals, the core use of evidence-based practices was highlighted as a feature of the professionalism portrayed by the role.

*“…be an accountable professional acting in the best interests of patients*,* by providing personalised psychological interventions that are evidence-based*,* compassionate and empowering.” (135 – CAP apprentice)*.

Finally, the CAP role was conceived to embed Considering the System (subtheme 5.4) in its approach. This consideration of systemic factors appeared to span varying levels of the system, including teamwork and collaboration (with both colleagues and service users as key stakeholders), the deployment of trauma-informed care principles, and in some services, an emphasis on practices informed and guided by community psychology.

*“Motivated to make positive change to systems and services. They bring an observer perspective to services and have a good value base so support positive change.” (55 - Supervisor)*.

## Discussion

This national survey on the CAP role identified five themes. These themes are: Widening Access to Psychology, Workforce development, Navigating the unfamiliar, Trained [Masters level] Professionals and An Emerging Ethos. Overall, a clear identity was identified, with CAPs depicted as offering versatile interventions in diverse health care settings, fostering a positive and encouraging integration of psychological expertise into the healthcare service. Within the rest of the discussion, each theme will be broadly considered in relation to CAP identity and implications and recommendations (see Table 3) for the role.

**Table 3:** Summary of recommendations based on the findings

Inclusion and access:
• Identifying and protecting a supervisory/apprenticeship training capacity within services.
Career framework:
• Guidelines outlining the respective skills, expertise and responsibilities of CAPs to highlight their status as trained professionals.
• Prioritize the management of progression and development of CAPs.
Professional engagement:
• CAP programmes to obtain BPS accreditation.
• CAPs to avail of the BPS Wider Psychological Workforce Register.
• CAPs to develop a collective voice, through regional CAP communities of practice.
• Employers and educational institutions to collaborate in developing a national strategy for CAPs, including a strategy for effective workforce planning.

### Widening Access to psychology

In Widening Access to Psychology, the CAP role is providing benefit to teams, clients and the CAPs themselves. Elevating psychological presence within teams can lead to better integration and collaboration, ultimately resulting in improved client care. Increased team cohesion and communication have been shown to benefit all parties involved [12]. Informal input and feedback through formulation are valued, although it is difficult to operationalise and quantify them as measurable outcomes [13]. Nonetheless, a strong psychological presence in teams may bolster team efficacy and overall job satisfaction [14].

### Workforce development

Diversity has become an important aspect in the workplace, emphasising the value of embracing one’s own identity and considering alternative perspectives. It also focuses on improving access for disenfranchised groups. The CAP role also demonstrates benefits for the people entering the profession by addressing known barriers [15]. A snapshot of demographics across CAP apprenticeship programmes in 2022 showed that people identifying as being from an ethnically minoritized background represented 41% [range: 16–71%] of those on programmes, and those identifying as having a disability represented 24% [range: 12–30%]. This overlaps with subthemes from Workforce Development and Navigating the unfamiliar, in the need to consider how we manage progression and develop the occupational identity. It is crucial that these areas are prioritised for attention and that the CAP apprenticeship does not foster a two-tiered psychological profession, with those from minoritized backgrounds presenting an occupational ceiling [16]. Cultivating a clear developmental trajectory towards a range of opportunities that align with the career aspirations of CAPs represents a step towards equity within the profession. Indeed, this aligns with the identified subtheme 1.3 “providing a route into the profession”, whereby some CAPs described the apprenticeship as a stepping stone towards occupational pursuits that have been otherwise inaccessible. Whilst the thematic structure also highlights a subtheme of “Supervision (2.4)” as a workforce challenge, it may be prudent for us to consider this challenge, going forward, alongside the wider aims of the psychological professions in fostering diversity amongst a historically homogenous professional group [17]. It may be that identifying and protecting a supervisory/apprenticeship training capacity in teams forms an important feature of a service or Trust level commitment to improved inclusion and access for those from marginalised groups.

The flexibility and versatility of the role (subtheme 2.3) is a crucial component, although services need to exercise caution. By allowing some degree of flexibility, this can ensure that our services are adaptable to local needs, aligning with the ambitions set forth in the NHS Long-term Plan [1]. The CAP role is developing within interprofessional teams, and as such, the identity will also be shaped by the role of others [18]. Nonetheless, the survey raises concerns about levels of responsibility and autonomy. Sufficient resources are necessary to support CAPs while also acknowledging the strain on practitioner psychologists as their roles expand and their time becomes increasingly dedicated to supervision rather than direct clinical work. There is a risk that this strain in the system places CAPs in a position of responsibility beyond their training and remit, with any emerging identity becoming confused with that of other established professionals. Characterising the work of CAPs as focusing on the delivery of psychologically informed formulation-driven practical interventions, while enabling clear opportunities for continuous professional development and progression, may help to protect the CAP identity and protect CAPs from overwhelming and inappropriate clinical responsibilities.

In relation to workforce development, respondents expressed their desire for the CAP role to possess a professional identity that offers tangible opportunities for growth. To continue attracting and retaining qualified individuals in the workforce, it is essential to establish a well-defined career framework and highlight the potential for progression within CAP roles. There is a clear need for a collaborative approach and a national strategy that specifically addresses the needs of CAPs. This may include showcasing success stories and providing examples of successful CAP roles post-qualification, as well as outlining clear paths for advancement within Trusts beyond the initial CAP role. Moreover, it is crucial that CAP programmes obtain accreditation from the BPS and that CAPs avail of the BPS Wider Psychological Workforce Register.

The NHS is currently experiencing a shortage of staff and resources, and while mental health is being prioritised, staffing levels have not increased accordingly [19]. There is evidence of a shortage of Clinical Psychologists in the UK, with a vacancy rate of 12% [20] and the role has been added to the UK Shortage Occupation List [21] NHS Health England (NHSE) has increased training provision; however, CAP funding falls outside of the NHSE remit and, as such, represents the initiatives of Trusts to address the issue. While addressing local needs, these Trust level initiatives are fragmented and do not provide a comprehensive solution to workforce pressure or support the development of the CAP occupation nationally. There is a need to match the working groups’ efforts, such as the trailblazer group and GTICAAP, with a national strategy for effective workforce planning.

### Navigating the unfamiliar

Indeed, amongst the theme Navigating the Unfamiliar, respondents highlighted a clear need for differentiating between roles, enhancing comprehension of the role, and establishing explicit expectations. Training providers, Trusts and accrediting bodies hold responsibility in guiding the development and integration of this novel professional identity, embedding it within existing structures and offering containment for CAP apprentices and qualified CAPs as they navigate this changing landscape of services. CAPs are highlighted by respondents as bringing a welcomed enthusiasm and energy that, supported and cultivated, brings an invaluable resource to teams. While the professional identity of a role is always evolving, it must be actively managed during times of change, and clear communication during periods of innovation is important, as these periods are often associated with insecurity and stress [18].

Amongst these efforts to provide a compass for the future (subtheme 3.4), CAPs should be encouraged and empowered to strive for a strong collective voice, which can be accomplished through the formation of CAP communities of practice within regional Psychological Professions Networks. Employers and educational institutions should continue to collaborate in developing a national strategy for CAP, as facilitated by the BPS Group of Trainers (GTICAAP) and the trailblazer group.

### Trained [Masters level] psychological professionals

Having trained [Masters level] psychological professionals in organisations provides a significant advantage. These individuals possess essential core skills that meet recognised BPS and apprenticeship standards. CAPs demonstrate a broad yet specialised (i.e., within their client group) knowledge, allowing them to effectively navigate complex environments while also assuming responsibility for their work. This expertise is reflected in their rigorous training, which demands a high level of commitment and expertise.

It is crucial to take into consideration the nature of CAP training programmes in a thoughtful manner. During training, both employers and universities need to consider and manage expectations while also acknowledging the demanding nature of the process. While some respondents suggested making the training process less strenuous by focusing on fewer but more meaningful assignments, there was also a request for a deeper understanding and teaching of various therapy modalities. This indicates a potential lack of comprehension about the role of CAPs, who are primarily trained to deliver interventions rather than therapy. While the flexibility of training programs to cater to local requirements is advantageous, CAPs were seeking a certain level of consistency in these programs nationwide. Guidance that allows for this should be provided by the regulating bodies (BPS, Ofsted and Institute for Apprenticeships & Technical Education), with national spaces such as the GTICAAP used to cultivate a broadly consistent offering, with sufficient flexibility to meet local needs. In maintaining quality of provision and to promote skill acquisition and practice, providers need to be supported by employers to promote quality in training as well as CAPs meeting service needs. There should be an awareness of factors that may affect the quality of delivery, such as workforce demand (case load prioritised over training), resources and funding for programmes [22] and the remote provision of training [23].

To address the uncertainties faced by both CAPs and their clinical supervisors, it is imperative that they are provided with clear guidelines outlining the respective skills, expertise and responsibilities of CAPs to highlight their status as trained professionals. Providers and Trusts can helpfully communicate this message through a commitment to providing both supervisors and teams with more comprehensive awareness, training, and education regarding the CAP role and value. The findings from this study can help contribute to that understanding, highlighting the key skills (subtheme 4.1) and specific contribution of CAPs summarised in Table 4.

**Table 4:** Specific aspects of the CAP role

1. Apprenticeship model: The CAP role is unique in that it is delivered as an apprenticeship model, which allows for local and service level variation in training and service delivery. [relates to (sub)themes 2, 2.2 and 2.3].
2. Specific area of clinical practice: CAPs gain experience and expertise in a specific area of clinical practice, such as adult mental health or intellectual disabilities. [relates to subtheme 4.2].
3. Bridging the gap: The CAP role bridges the gap between assistant psychologists and clinical psychologists, providing specialist interventions such as third wave CBT. [relates to subtheme 1.2].
4. Masters level training: CAPs gain a masters level qualification through their training. [relates to theme 4]
5. Flexibility: The CAP role allows for flexibility in the use and application of psychological knowledge in a non-prescriptive way. [relates to subthemes 2.3 and 4.1]
6. Focus on practical interventions: The CAP role emphasises practical interventions rather than theoretical knowledge. [relates to subthemes 2.3, 4.1 and 4.2]
7. Formulation-driven development: The CAP role offers an applied, formulation-driven development opportunity that is not model specific. [relates to subthemes 1.2 and 2.3]
8. Autonomy and supervision: CAPs are able to work semi-autonomously under the supervision of a clinical psychologist. [relates to subthemes 2.4 and 4.3]
9. Specialist to one area of practice: CAPs are trained to work with one client population and gain a wealth of experience in working with this one population throughout their training. [relates to subtheme 4.2]

CAPs are accountable and responsible for their assigned tasks and have to work to standard of professional accountability (as recognised by being on the BPS Register, which in turn is an accredited register of the Professional Standards Authority). While not regulated by law (as with the Health & Care Professions Council register), it is important for CAPs to recognise and adhere to their level of competence, as determined by their clinical supervisor. Any attempt to exceed this level may be considered negligent. CAPs operate within the applied psychology pathway and have work roles and skills beyond the delivery of specific therapies and therefore are placed within the practitioner psychologist supervisory framework. For all psychologists, established supervisory structures are in place to aid in assessing and maintaining the required level of competence applicable to the role [24]. It is therefore concerning to see a requirement for supervision either questioned or not routinely available to CAPs.

CAPs provide a post-graduate, trained, competent solution to gaps in the workforce, utilising the apprenticeship model, but it is necessary to contrast this approach with other initiatives introduced by the NHSE. To preserve CAPs’ unique identity, we must ensure their presence by both establishing and supporting comprehensive training programmes nationwide, which in turn requires CAPs to be factored into long-term workforce planning rather than plugging short-term gaps. The training process, and specifically the demands of apprenticeship training, were highlighted by respondents and should be noted by trainers, employers and prospective CAPs alike. A responsiveness to hearing concerns, recognising where processes, structures, and demands may cause overwhelming or inappropriate burden during a period of learning and consolidation, is essential to the well-being and longevity of this emerging workforce [25]. Where attracting talented and valued staff is essential to the stability and growth of the profession, proactive action to prevent and tackle burnout - as CAPs juggle the demands of clinical work in the NHS alongside post-graduate academic study - is crucial. Dialogue between employers and training providers is paramount in ensuring a workload and a job plan that allows for training needs to be met, balanced with meeting service needs [26]. Dedicated forums whereby the CAP voice can be heard and listened to - such as student feedback spaces within training institutions - are valuable opportunities to monitor the establishment of manageable and sustainable training demands.

### An emerging ethos

Clear values are highlighted in the theme ‘emerging ethos’. The CAPs have developed proficient therapeutic skills, displaying cultural competence and valuing lived experience, aspects highlighted as being deficient in clinical programmes [27]. They are reflective practitioners who take into consideration both the systems they operate within and those of their clients. These professionals are dedicated, diligent, and showcase resilience and adaptability in their work. They adhere to an evidence-based approach, ensuring responsibility and accountability.

The question of establishing identity and the concerns related to this mirror those experienced by clinical psychology in the past [28]. While psychologists have formed part of the NHS workforce since its inception, their role has evolved from psychometricians assisting psychiatrists with diagnosis to scientist-practitioners providing a broad range of functions within the NHS [29, 30]. Clinical Psychology, as a broad and pragmatic profession informed by humanistic and scientific values, has laid the foundations for the development of CAPs, but as a new occupation within the NHS, the CAP role will need to evolve in its own right.

Indeed, thus far, the CAP identity and ethos appear to be characterised by a commitment to their own professionalism, to the relationship with their clients, and consideration of wider systems. This latter point represents a relevant shift towards principles aligned with community psychology approaches and aligns with the NHS Long Term Plan [1] in its acknowledgement of local communities and the impact of social circumstances with regard to psychological health. Linking with the subthemes relating to widening access to services for clients and important considerations of diversity, this emerging CAP ethos may offer an opportunity to embed care that extends beyond the traditional ‘therapy’ of mental health services towards something that meets the bespoke needs of local and diverse populations.

This is the first study investigating the CAPs as an occupation. While representation across regions was variable, the study included a large sample with diverse perspectives on the CAP identity, and the findings have transferability for other practice settings implementing CAP apprenticeship programs nationwide. Professional identity is shaped by tasks, skills, and individuals’ emotional and existential journeys [31]. The initial framework in this study emphasized vocational aspects, potentially overlooking the construct’s complexity. Personal satisfaction, moral and ethical fulfilment, and the alignment of one’s work with their personal identity are crucial factors in forging a professional identity. Affective elements influence how individuals perceive their profession and their place within it, shaping personal outcomes and professional culture. Future research should employ frameworks emphasizing the complexity of professional identity formation as this role evolves over time [31].

## Conclusions

The success of this new role relies heavily on the fact that the CAP is a response to an identified need rather than a surplus workforce waiting for deployment. It is a pioneering group that has been developed on-site to meet locally identified workforce needs. The nation faces significant challenges in health and social care, such as an aging population with increasing health and social care needs and children and young people who require more mental and physical health support than ever before. A fresh approach to healthcare is necessary, one that acknowledges emotional and psychological requirements as essential components of overall wellbeing. CAPs present unique opportunities to meet these challenges.

### Electronic supplementary material

Below is the link to the electronic supplementary material.

Supplementary Material 1

## Data Availability

The dataset used and/or analysed during the current study are available from the corresponding author on reasonable request.

## Footnotes

1 The role of the CAP was initially created in response to a shortage of clinical psychologists in Cornwall. Cornwall Partnership NHS Foundation Trust and Exeter University developed a course based on the Scottish model, training a first cohort in 2018. This model served as the basis for the CAP apprenticeship, developed through a stakeholder ‘trailblazer’ group (including NHS Trusts, Voluntary, Community, and Social Enterprise organisations, Universities, the Institute for Apprenticeships and Technical Education (IATE), and the British Psychological Society (BPS)) who conducted a nationwide public consultation. Following this the IATE [2] approved an occupation standard for the CAP apprenticeship.

## Abbreviations

CAP: Clinical Associate in Psychology
NHS: National Health Service
BPS: British Psychological Society
CMHT: Community Mental Health Team
CAMHS: Child & Adolescent Mental Health Services
HTT: Home Treatment Team
GTICAAP: Group of Trainers in Clinical Associates in Psychology
NHSE: NHS England
