# Supplementary material for: The identity of clinical associates in psychology: a cross sectional, national survey
Source: BMC Med Educ. 2024 Jul 31;24:825. doi: 10.1186/s12909-024-05802-7 (PMC11293191; doi:10.1186/s12909-024-05802-7)
Supplement: Supplementary file 1 — Supplementary Material 1 [file 12909_2024_5802_MOESM1_ESM.docx]

Supplementary Materials.

| Please write a word or phrase that you associate with the CAPs role in each box. Please follow your first thoughts. |
| --- |
| Can you tell me more about what you've put here [participants phrase provided]? Write as if explaining to someone who does not know the role. |
| How do you think the CAP role compares to other roles in mental health provision? |
| What do you think are the main strengths of the CAP role? |
| What is unique about the CAP role? |
| What are the common skills and knowledge required for the CAP role? |
| What are the common risks and challenges associated with the CAP role? |
| What are the common values and attitudes associated with the CAP role? |
| What do you think could be done to improve the delivery of the CAP role? |

Table 1. Survey Questions.
